# Altered Intrinsic Functional Connectivity in Language-Related Brain Regions in Association with Verbal Memory Performance in Euthymic Bipolar Patients

**DOI:** 10.3390/brainsci3031357

**Published:** 2013-09-12

**Authors:** Britta Reinke, Vincent van de Ven, Silke Matura, David E. J. Linden, Viola Oertel-Knöchel

**Affiliations:** 1 Laboratory of Neurophysiology and Neuroimaging, Department of Psychiatry, Psychosomatic Medicine and Psychotherapy, Goethe University, Heinrich-Hoffmann-Str. 10, Frankfurt 60528, Germany; E-Mails: britta.reinke@arcor.de (B.R.); Silke.Matura@kgu.de (S.M.); 2 Department of Cognitive Neuroscience, Faculty of Psychology and Neuroscience, Maastricht University, Maastricht 6211 LK, The Netherlands; E-Mail: v.vandeven@maastrichtuniversity.nl; 3 MRC Centre for Neuropsychiatric Genetics & Genomics, Institute of Psychological Medicine and Clinical Neurosciences, School of Medicine, Cardiff University, Cardiff CF10 3AT, UK; E-Mail: LindenD@cardiff.ac.uk

**Keywords:** resting-state fMRI, bipolar disorder, auditory network

## Abstract

Potential abnormalities in the structure and function of the temporal lobes have been studied much less in bipolar disorder than in schizophrenia. This may not be justified because language-related symptoms, such as pressured speech and flight of ideas, and cognitive deficits in the domain of verbal memory are amongst the hallmark of bipolar disorder (BD), and contribution of temporal lobe dysfunction is therefore likely. In the current study, we examined resting-state functional connectivity (FC) between the auditory cortex (Heschl’s gyrus [HG], planum temporale [PT]) and whole brain using seed correlation analysis in *n* = 21 BD euthymic patients and *n* = 20 matched healthy controls and associated it with verbal memory performance. In comparison to controls BD patients showed decreased functional connectivity between Heschl’s gyrus and planum temporale and the left superior and middle temporal gyrus. Additionally, fronto-temporal functional connectivity with the right inferior frontal/precentral gyrus and the insula was increased in patients. Verbal episodic memory deficits in the investigated sample of BD patients and language-related symptoms might therefore be associated with a diminished FC within the auditory/temporal gyrus and a compensatory fronto-temporal pathway.

## 1. Introduction

The classical Kraepelinian dichotomy of major psychotic disorders into dementia praecox (later termed schizophrenia), a disease of relentless progression with prominent cognitive abnormalities, and manic depression, a cyclic disorder without progressive loss of cognitive functions, is increasingly being challenged on clinical and biological grounds [1]. One of the central questions is whether schizophrenia and manic depressive or bipolar disorder (BD) are parts of a spectrum of disorders with varying cognitive, affective and other psychopathological deficits. In both disorders, dysfunctions in language-related memory such as verbal episodic memory have been reported and may be affected during both acute episodes and remitted state (schizophrenia: [2]; bipolar: [3,4,5,6,7]). The investigation of the neural structures underlying language-related cognitive abilities is of interest in order to find possible direct links between the clinical symptomatology and underlying neuronal circuits. We therefore investigated whether language-related memory performance in BD is related to a brain network that is involved in language and speech perception.

The main brain network that is known to be involved in speech perception is located in the temporal and parietal lobes of the dominant hemisphere, particularly the planum temporale (PT), posterior part of the middle temporal gyrus and the temporo-parietal junction [8]. The superior temporal cortex as main language and speech brain area is known to be functionally and structurally asymmetric, and in most healthy right-handers left Heschl’s Gyrus (HG) and PT are larger than its right hemisphere counterpart [9]. In schizophrenia patients, functional and anatomical brain imaging studies have shown reduced PT hemispheric asymmetry [10,11], which may be associated with individual psychopathology [10,12]. Structural imaging findings in BD indicate a volume loss of the superior temporal gyrus [13]. So far, no direct link has been established between volume reductions in HG and PT and individual psychopathology [14]. However, [15] reported that the anterior portion of the left PT of acute manic BD patients was comparable to those of SZ patients, but thinner in comparison with healthy controls. The findings of [15] are of interest, as they suggest that the structural alterations in BD within the superior temporal cortex may be asymmetric.

Temporal lobe function and its laterality can be investigated with imaging and psychophysical techniques [16]. Some studies have suggested that functional asymmetry is reduced in affective disorders [17,18,19,20], as evidenced, for example, by an altered right ear advantages on dichotic listening experiments [17]. The authors of [21] reported that BD patients showed abnormal laterality of the primary auditory cortex using MEG (magnetoencephalography). However, no other study has investigated the functional connectivity of the auditory network in BD patients.

The aim of the current study was to investigate the functional connectivity (FC) within the auditory network in BD patients by resting-state fMRI. Resting-state fMRI displays a novel approach to investigate the brain's intrinsic neuronal activity in the absence of a distinct stimulation or task. The functional connectivity between different brain areas describes temporal relations between the activation patterns of these areas and thereby is assumed to reflect their functional communication. It is thereby assumed that areas with a high correspondence in neuronal activity are functionally linked. In psychiatric research it can be used to assess neuropathological alterations associated with the clinical picture [22]. Compared to task-related fMRI the advantage of resting-state fMRI, as employed in the current study, is that the underlying functional connectivity of brain areas is accessible independently of an experimental paradigm. The existing resting-state studies in BD focused on frontal and limbic seeds [23,24,25,26] or on the so-called default mode network (DMN) [25,27].

We sought to investigate whether the auditory network shows abnormal functional connectivity in BD. We selected the HG and PT, which are key components of the auditory cortex and the auditory speech perception [8] and thus could be associated with the symptomatology of BD patients, particularly verbal memory deficits. Furthermore, we investigated whether temporal lobe aberrant functional connectivity in BD may be directly associated with language-related memory functioning. We expected a diminished functional connectivity in the group of BD. Furthermore, we expected significant relationships between the functional connectivity of auditory brain regions and the performance in an episodic memory task with auditorily presented verbal stimuli. 

## 2. Results and Discussion

### 2.1. Cognitive Testing and Individual Psychopathology

The scores of the BDI II (Beck Depression Inventory [28]) were significantly different between patients and controls (t (39) = 3.64, *p* = 0.001), but there was no group difference regarding the BRMAS (Bech Rafaelsen Manie Scale [29]) scores (*p* > 0.05). Further tests for group differences showed no significant differences between the groups for the negative affect (NA) of the PANAS (Positive and Negative Affect Schedule [30]; *p* > 0.05), but there was significantly higher positive affect (PA) in the control group (t (39) = −4.84, *p* < 0.01). 

Controls performed significantly better than BD patients in CVLT’s (California Verbal Learning Test [31]) subscales *learning sum*, *delayed free recall I* and *delayed free recall II* (CVLT LS: t (39) = −2.73, *p* = 0.01; CVLT DFR I: t (39) = −3.06, *p* = 0.004; CVLT DFR II: t (39) = −2.26, *p* = 0.031). In the in the *yes/no recognition* performance there was a trend to a better performance of controls in comparison to BD patients (CVLT YNR: t (39) = −1.74, *p* = 0.092; see Table 1).

### 2.2. Seed Region: HG Bilateral

The ANCOVA with age, sex and education as nuisance covariates was corrected using FDR as correction for multiple comparisons thresholded at *q* ≤ 0.05 (view statistical analysis). The multi-subject result map (voxel-by-voxel one-sample *t*-test of connectivity values) with voxel clusters of HG bilateral functional connectivity included bilateral parts of the frontal and temporal gyrus, the insula, lingual gyrus and the caudate.

**Figure 1:**
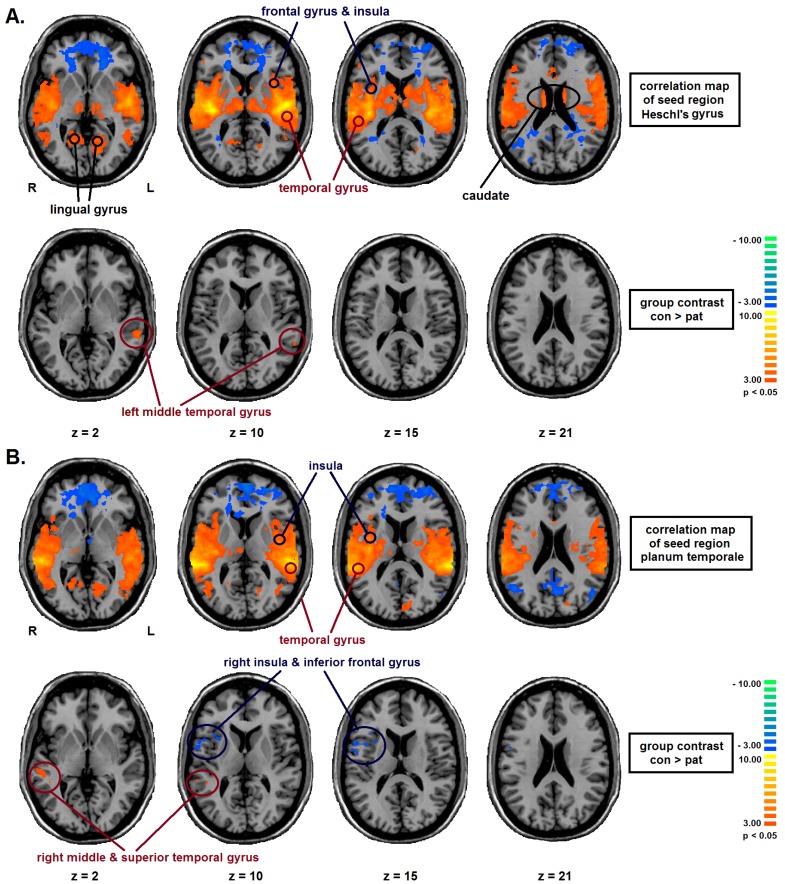
One-way ANCOVA of the functional connectivity scores (FC) with Group as between-subject factor and age, sex and education as covariates (ANCOVA F-map corrected for FDR and cluster size) and *T*-Tests of the group contrast controls (*n* = 20) *vs*. bipolar disorder (BD) patients (*n* = 21), cluster threshold: *p* < 0.05). The upper rows indicate the functional connectivity maps in the entire sample, the lower rows the group contrast. (**A**): Significant regions with bilateral Heschl’s Gyrus (HG) as seed-region. Colour code: red = positive FC. blue = negative FC. (**B**): Significant regions with bilateral planum temporale (PT) as seed-region. The left side in the figure indicates the right side of the brain (radiological convention). Colour code: red = CON > PAT. blue = PAT > CON.

**Figure 2:**
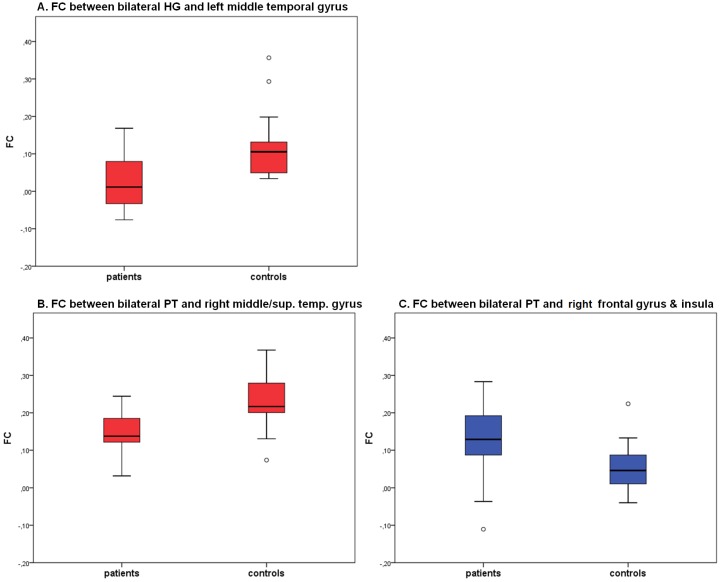
Coefficients of functional connectivity (FC) of significant areas in the group comparisons of the seed-regions for BD patients (*n* = 21) and controls (*n* = 20). Upper rows (**A**): FC between bilateral Heschl’s Gyrus (HG) and left middle temporal gyrus. Lower rows (**B**, **C**): FC between bilateral planum temporale (PT) and right middle and superior and middle temporal gyrus and between bilateral PT and the right inferior frontal and precentral gyrus and right insula. red = patients < controls, blue = patients > controls. Vertical lines indicate the upper and lower quartiles, single points display outliners.

*Post-hoc* pairwise comparisons (two-sample *t*-tests) using cluster level thresholding as correction for multiple comparisons thresholded at *p* ≤ 0.05 showed that functional connectivity between bilateral HG and left middle temporal gyrus (BA 22 (Brodmann area)) was significantly decreased in BD patients in comparison with healthy controls (view Figure 1A, Figure 2A, Table 2).

**Table 1:** Sociodemographic characteristics and cognitive performance of the patient group (PAT; *n* = 21) and the control group (CON; *n* = 20). Standard deviations (SD) are in brackets. MWT-B = Mehrfachwahl-Wortschatz-Test, BDI = Beck Depression Inventory II, BRMAS = Bech Rafaelsen Manie Scale, PANAS = Positive and Negative Affect Schedule (PA = positive affect, NA = negative affect), CVLT = California Verbal Learning Test (LS = Learning sum, DFR I = delayed free recall I, DFR II = delayed free recall II, YNR = yes/no recognition).

	PAT Mean (SD)	CON Mean (SD)	Significance
**Number**	21	20	-
**Gender**	9 f/12 m	8 f/12 m	χ^2^ = 0.03, *p* = 0.55
**Age (years)**	35.67 (10.68)	36.90 (11.06)	t = 0.36, *p* = 0.72
**MWT-B**	29.86 (3.31)	31.80 (2.98)	z = −1.83, *p* = 0.06
**Handedness**	all right handed	all right handed	-
**BDI**	9.85 (9.00)	2.00 (3.57)	t = 3.64, *p* = 0.001
**BRMAS**	0.38 (0.59)	0.25 (0.44)	t = 0.80, *p* = 0.43
**PANAS PA**	25.62 (8.34)	35.75 (4.61)	t = −4.84, *p* < 0.01
**PANAS NA**	15.71 (5.25)	14.20 (4.01)	t = 1.03, *p* = 0.31
**CVLT LS**	53.90 (10.27)	61.45 (7.21)	t = −2.73, *p* = 0.01
**CVLT DFR I**	11.29 (2.76)	13.55 (1.88)	t = −3.06, *p* =0.004
**CVLT DFR II**	12.33 (3.02)	14.05 (1.70)	t = −2.26, *p* = 0.031
**CVLT YNR**	15.10 (1.14)	15.60 (0.68)	t = −1.74, *p* = 0.092

### 2.3. Seed Region: PT Bilateral

The multi-subject result map (voxel-by-voxel one-sample *t*-test of connectivity values) with voxel clusters of PT bilateral functional connectivity included parts of the bilateral superior and middle temporal gyrus and the bilateral insula (FDR ≤ 0.05).

*Post-hoc* pairwise comparisons (two-sample *t*-tests) showed that compared to healthy controls functional connectivity of BD patients was significantly decreased between the bilateral PT and the right superior and middle temporal gyrus (BA 22), next to the seed region (view Figure 1B, Figure 2C,D, Table 2). Furthermore, in comparison to controls, euthymic bipolar patients showed an increased functional connectivity between bilateral PT and a frontal cluster (BA 13 & 44) including the right inferior frontal and precentral gyrus as well as the right insular cortex (cluster level thresholded, *p* ≤ 0.05; view Figure 1B, Figure 2B,C, Table 2).

**Table 2:** *Post-hoc* group comparisons between controls (CON; *n* = 20) and bipolar patients (PAT; *n* = 21) in the functional connectivity pattern between bilateral seed regions of Heschl’s Gyrus (HG) and planum temporale (PT) and whole-brain functional connectivity. BA = Brodman area, TAL = Talairach coordinates, FC = functional connectivity scores.

Seed-Region	Area	BA	TAL-Coordinates	Nr of voxels	group	FC	T	*P*
bilateral HG	center middle temporal gyrus	22	−58, −36, 5	929	CON	0.12 (0.09)	3.94	<0.01
					PAT	0.02 (0.07)		
bilateral PT	right middle & superior temporal gyrus	22	51, −35, 4	949	CON	0.23 (0.07)	4.35	<0.01
					PAT	0.14 (0.06)		
	right inferior frontal gyrus, precentral gyrus, insula	13, 44	45, 7, 11	1406	CON	0.05 (0.06)	−3,20	<0.01
					PAT	0.13 (0.10)		

### 2.4. Correlation Analysis

#### 2.4.1. Individual Psychopathology

In the patient and control group there was no significant correlation between the current affect (PANAS PA/NA) and the degree of functional connectivity to the auditory cortex in the investigated clusters. Furthermore, there were no significant correlations between the measures of individual psychopathology (BDI II, BRMAS) and the subjects’ functional connectivity.

#### 2.4.2. Cognitive Performance

After correcting for multiple testing, there were no significant correlations between the identified correlation coefficients of functional connectivity and the performance in the verbal episodic memory task (CVLT).

Nonetheless, there are two interesting significant correlation coefficients not corrected for multiple testing we would like to report (view Table 3). They show a trend towards a relationship between subjects functional connectivity and their verbal memory performance. In BD patients a lower functional connectivity between the right middle and superior temporal cluster and bilateral PT was accompanied by a better performance in verbal episodic memory. Patients’ functional connectivity between these areas correlated significantly negative with the learning sum (LS) in the CVLT (CVLT LS: *r* = −0.477, *p* = 0.029). Furthermore, control subjects showed a significant negative correlation between fronto-temporal functional connectivity, which was increased within the patients’ group, and the recognition performance of the CVLT. A lower fronto-temporal functional connectivity was accompanied by a higher performance in the yes/no recognition (YNR) task (CVLT YNR: *r* = −0.479, *p* = 0.033).

**Table 3:** Significant correlations (Pearson Product Moment Correlation, two-tailed) in the bipolar patients (PAT) and control group (CON) regarding all significant areas during resting-state analysis and the cognitive performance. The table contains all comparisons which deemed significant in the bipolar patients or control group separately without correcting for multiple testing. CVLT = California Verbal Learning Test (LS = Learning sum, YNR = yes/no recognition). All tests which showed no significant differences across the correlation analyses were excluded from the table due to space limitation.

Seed-Region	Area	group	Significant correlations
bilateral HG	center middle temporal gyrus	CON	-
		PAT	-
bilateral PT	right middle & superior temporal gyrus	CON	-
		PAT	CVLT LS: *r* = −0.477, *p* = 0.029
	right inferior frontal gyrus, precentral gyrus, insula	CON	CVLT YNR: *r* = −0.479, *p* = 0.033
		PAT	-

#### 2.4.3. Medication

To control for possible effects of medication on functional imaging results a medication index [32] and the duration of taking psychopharmacological medication were assessed. There were no significant relationships between the indices of medication and the results in resting state neuronal activation (*p* > 0.05).

### 2.5. Summary of Results

Compared to healthy controls, the functional connectivity of BD patients was significantly decreased between the auditory cortex and left- and right-sided clusters within the temporal gyrus (superior and middle temporal gyrus). Additionally, there was an interesting pattern of group differences in fronto-temporal functional connectivity. In comparison with healthy controls patients’ functional connectivity was significantly increased between bilateral HG and right precentral/inferior frontal gyrus and insula.

The results indicate that BP showed a diminished functional connectivity within the temporal gyrus and auditory cortex areas. Decreased cognitive functioning in tasks requiring auditory and speech perception might thus be accompanied by neurophysiological alterations in relevant brain areas. As there is to date no study examining PT and HG functional connectivity in BD, we are the first group that reports altered functional connectivity within the temporal gyrus and auditory cortex areas. These findings are compatible with those reported for schizophrenia [33,34,35]. This is of interest for current pathophysiological models of BD because it indicates that BD and SZ share alterations within the auditory network which may be linked with clinical symptoms.

Furthermore, there seems to be a dysbalance in patients’ functional connectivity between the auditory cortex and the right frontal cortex. Functional connectivity between the seed region in planum temporale and a cluster in the right-sided inferior frontal and prefrontal gyrus and the insula was increased in the group of BD patients. The higher correlation of neuronal activity at rest between bilateral PT and right-sided frontal gyrus may thus display a compensatory mechanism to reserve fronto-temporal functioning and a diminished recruitment of the auditory cortex in the patient group.

### 2.6. Association with Language-Related Memory Performance

Both seed regions belong to the auditory cortex which is a crucial node for verbal episodic memory tests with auditorily presented stimuli like those in the CVLT. In the CVLT patients’ performance was significantly lower in the measures of immediate and delayed free recall than the memory performance of control subjects. Thus, temporal lobe dysfunction in terms of a diminished functional connectivity within the temporal/auditory cortex might affect the encoding and recall of verbal episodic memory and contribute to verbal episodic memory deficits often found in remitted BD patients [3,4,5,6,7]. Differently from what we expected, there were no significant correlations between the values of functional connectivity and the performance in the verbal episodic memory task. Nonetheless, our results support previous findings of a disruption of functional connectivity patterns in bipolar patients [23,24,25,26,27] and display these for important structures of the auditory cortex system. Even though we did not find a direct link between subjects’ functional connectivity and their episodic memory performance, a disruption of connectivities in temporal/auditory structures is an interesting finding in the investigation of the neurophysiological basis of bipolar disorder. It could play an important role in an altered speech and language perception and language-related symptoms patients suffer of.

There are two interesting correlation coefficients which reached the level of statistical significance without correcting for multiple testing and thereby indicate a trend towards a relationship. Of course, these might be false positive results. On the other hand, they might display a hint for an existing relationship and be significant if we had a bigger sample size. Because of this, we wanted to report and discuss them, emphasizing again that they are not corrected for multiple testing and only show a trend. Even though patients' functional connectivity within the temporal gyrus, which is known to subserve language and speech function, was decreased, there was an inverse relationship with their episodic memory performance. A lower functional connectivity between bilateral PT and the right middle and superior temporal gyrus was significantly accompanied by better verbal episodic recall. This inverse relationship was unexpected but there are other occasional reports in the literature of associations of decreased connectivity with better cognitive performance [36]. A possible explanation for this unexpected relationship could be a general disruption of temporal connectivity pathways causing inverse relationships between functional connectivity and verbal memory performance in the patients’ group. Additionally, it might be possible that patients use compensatory pathways, e.g., fronto-temporal, to preserve memory function while decreasing ineffective connectivity within the temporal gyrus. In this case, a lower connectivity within the distinct temporal pathways we investigated could serve as a strategy to provide a more effective functioning in compensatory pathways. This idea of the use of compensatory pathways is in line with our results regarding fronto-temporal functional connectivity. Functional connectivity between bilateral PT and the cluster within the right inferior frontal and prefrontal gyrus and the insula was significantly increased in patients compared to healthy controls. Furthermore, control subjects showed a negative relationship between fronto-temporal functional connectivity and the recognition task of the CVLT. A higher fronto-temporal functional connectivity was accompanied by lower cognitive performance. A possible interpretation of this relationship is that the use of compensatory pathways is due to a diminished connectivity within the auditory/temporal cortex and thus associated with a lower verbal memory performance. Even though we assume these mechanisms occur in the clinical population of BD patients they may also occur in non-clinical populations and display a possible vulnerability for memory deficits or even affective disorders.

### 2.7. Association with Individual Psychopathology

Because we investigated the psychopathology of euthymic outpatients without acute symptoms of mania or depression we did not expect broad differences in the scores of psychopathology between patients and controls. Nonetheless subclinical symptoms can be present even in the state of remission. This can be seen in the significant higher level of depression and lower level of positive affect present in the patients’ group and was thereby expected.

Because of the subclinical symptoms seen in increased levels of depression and decreased levels of positive affect, we expected corresponding correlations between patients’ symptomatology and their functional connectivity. Nonetheless, in the patients’ group there were no significant correlations between the functional connectivity of the investigated clusters to the auditory cortex and their current psychopathological state. This may be due to pathological mechanisms in the patients’ group disrupting expected relationships. Furthermore, we investigated a group of outpatients who were not suffering from acute symptoms. The lack of association between functional connectivity and individual psychopathology may thus be explained by the fact that altered patterns of functional connectivity display a trait marker of bipolar disorder which is independent of acute and subclinical symptoms.

### 2.8. Limitations

As the overall number of resting-state studies in BD patients is relatively small [23,24,25,26,27], direct comparisons between these studies are difficult. Furthermore, the sample selection is different, e.g., concerning whether acute or remitted BD patients were assessed. Also, none of the existing resting-state studies directly addressed auditory network functional connectivity. Furthermore, in order to establish associations with psychopathology, it would be necessary to extend the current study design to patients who are acutely ill.

However, in the current study, the number of participants per group is sufficient compared with other functional imaging studies, the groups were well matched and the seed-regions are probed in other imaging studies. Also, the pattern of results fits into current pathophysiological models of BD. 

Regarding the interpretation of correlation coefficients, again it should be emphasized that we did not find any significant correlations after correcting for multiple testing. The two correlations between functional connectivity and episodic memory performance we reported are not corrected for multiple testing, and might thus be false positives only showing a trend towards a significant relationship.

## 3. Experimental Section

### 3.1. Participants

We examined 21 outpatients with bipolar disorder (mean age M = 35.67 years (SD = 10.68)) who were diagnosed according to the DSM-IV criteria for BD [37] and 21 matched (age, gender, education) controls (M = 36.90 years (SD = 11.06)). We ensured that both groups did not differ significantly in the general intelligence, measured with the MWT-B (Mehrfachwahl-Wortschatz-Test [38], the German equivalent of the “Spot-the-Word test”) (ANOVA, *F* (1) = 3.889, *p* > 0.05). Participants were provided with a description of the study and gave written informed consent before participating. Experimental procedures were approved by the ethical board of the medical department of the Goethe-University, Frankfurt/Main, Germany. The patients and controls also performed other imaging experiments of our working group.

The patients were measured during remitted episodes, ensured using the BDI II (Beck Depression Inventory; [28]) for depressive symptoms and BRMAS (Beck Rafaelsen Mania Scale; [29]) for manic symptoms and there cut-off scores defining a clinical relevant episode (remitted state: BDI II scores of <18, BRMAS scores of <7) as well as the DSM-IV criteria for acute episodes. None of the patients fulfilled any of the cut-off scores for an acute episode. Medication status was also assessed using the formula by [32] to compute equivalent scores for mood stabilizers. It was ensured that all BD patients were in stable medication at a minimum of 4 weeks.

Controls were excluded if they were currently abusing drugs, had any neurological disease, any history of psychiatric disorders including Axis I and Axis II disorders according to DSM-IV [37], and an inability to provide informed consent. We ensured that none of the controls had any positive family history of affective or psychotic disorder using the Structured Clinical Interview for DSM-IV (SCID-I and SCID-II; German version: [39] (see Table 1 for further details). All probands were right-handed and german native speakers.

### 3.2. Cognitive Testing

In order to assess episodic memory performance, we conducted the California Verbal Learning Test (CVLT; [31]), which includes a learning, an interference and a recognition list, which are presented auditorily by the test administrator. We used the following parameters of the CVLT assessment: *Learning sum* (*LS*), *delayed free recall I* (DFR I) and *delayed free recall II* (DFR II), *recognition discriminability* (D). The *learning sum* displays the number of correctly recalled items during five learning trials, the delayed free recall the number of recalled items after presentation of the interference list (*delayed free recall I*) and after a delay of 20 min (*delayed free recall II*). The *yes/no recognition* performance assesses the identification of correct words (hits) and wrong words (distractors) of the recognition list.

### 3.3. Individual Psychopathology

Current individual psychopathology was assessed using the German version of the Beck Depression Inventory II (BDI II; [28]) and the German version of the Bech Rafaelsen Manie Scale (BRMAS [29]). All participants were also screened for their current mental state using the Positive and Negative Affect Schedule (PANAS; [30]). For the PANAS, two scores were computed: the *positive affect* (PA) and the *negative affect* (NA). In the BDI II and BRMAS a higher score corresponds to more severe symptoms. Regarding the PANAS, higher scores represents a higher positive (PANAS PA) or negative affect (PANAS NA).

### 3.4. Imaging: Data Acquisition

All participants underwent a resting-state functional imaging sequence (EPI-sequence, 400 volumes, voxel size: 3 × 3 × 3 mm^3^, TR = 2000 ms, TE = 30 ms, 33 slices covering the whole brain, slice thickness = 3mm, distance factor = 20%, flip angle = 90 degrees) and a high-resolution T1-weighted anatomical measurement (MDEFT sequence [40]; 176 slices, 1 × 1 × 1 mm^3^) on a Siemens Magnetom Allegra 3 Tesla MRI system (Siemens Medical Systems, Erlangen, Germany) at the Goethe University Brain Imaging Center, Frankfurt am Main, Germany. 

The session started with the resting-state functional measurement, where participants were instructed to lie still and look at a white fixation cross, presented in the centre of the visual field. Participants were scanned with dimmed lights, and they were instructed not to engage in any overt speech during the scanning sequences. This lasted eight minutes and was followed by the anatomical scan described above. The anatomical MRI scans of all participants were reviewed by a neuroradiologist who did not find any underlying pathology.

### 3.5. Imaging: Data Preprocessing

Functional imaging data were preprocessed and co-registered to the anatomical MR images using the BrainVoyager QX software version 2.2 [41]. First, all functional scans were corrected using the slice-time correction, rigid-body motion correction (Levenberg-Marquardt algorithm), linear trend removal and high-pass temporal filtering (3 cycles per time course, cutoff = 0.0075 Hz). Images with more than 2 mm of motion or severe visual motion artifacts were removed from runs. Furthermore, the six head movement parameters from the motion correction procedure were considered as covariates in the further analysis (see below). Additionally, we correlated the maximal amount of motion with functional connectivity to exclude an impact of head motion on our results and did not find any significant relationships. Afterwards, three-dimensional (3D) anatomical scans were transformed into Talairach space [42] using a 12-point affine transformation. We used automated routines of the BrainVoyager software to co-register the functional data to the anatomical scans of each participant. At least, the functional data were resampled to an iso-voxel size of 3 × 3 × 3 mm^3^.

### 3.6. Imaging: Statistical Analysis

We conducted a seed correlation analysis (SCA), using bilateral Heschl’s gyrus and planum temporale as seed-regions. The seed-regions were anatomically defined, using the anatomical scans of all participants and following the landmarks described by [43,44]. Initial identification of HG and PT was achieved using the vmr-segmenter by [45] or [46]. HG and PT gray matter was traced on coronal images to the end of the Sylvian fissure, and the gray matter of the ascending ramus of the Sylvian fissure was also included. After drawing, HG and PT ROIs could be viewed in any plane and as a 3-dimensional object for further editing. All sagital MRIs were manually checked to confirm HG and PT boundaries. The location of PT and HG and the steps to create seed-regions were similar to the study by [10]. The final bilateral ROIs were used to examine functional connectivity during resting-state using the Matlab software (MathWorks, Natick, MA, USA) and freely available toolboxes and custom-written routines developed by our working group (Vincent van de Ven).

During SCA, the functional time-series of one or more pre-defined brain areas (=seed regions) are sampled and correlated with all other functional time-series. We corrected the seed time-series for potential nuisance variables (Z-normalized), which included fMRI signal from ventricles, white matter, the global (whole-brain) signal, and the six head movement parameters from the motion correction procedure as described by [47,48]. All predictors were included into a two-level general linear model (GLM) [33,49,50]. Functional connectivity coefficients for each participant were estimated using the GLM and corrected for nuisance variables (described above). For the first-level analysis, the time-series from the voxels that were tagged by the HG and PT VOIs were averaged and standardized (Z-normalization) within each VOI. Second-level analyses of covariance (ANCOVA) for bilateral HG and bilateral PT separately included group as between-subject factor and age, sex and education as nuisance covariates.

Results of the group comparisons were visualized on the anatomical images using the BrainVoyager QX software. We used a statistical threshold of *q* < 0.05 (corrected for multiple comparisons using cluster size thresholding [51]). Using the regions of effects that survived the visualization thresholds, we defined regions-of-interest (ROIs) and computed voxel functional connectivity coefficients from these ROIs for each participant. The functional connectivity coefficients were used as dependent variables for two-tailed *post-hoc* two-sample *t*-tests.

### 3.7. Correlation Analyses

We calculated correlation coefficients in order to assess the relationship between HG and PT functional connectivity and individual psychopathology (BDI II, BRMAS, PANAS; Spearman rank, rho) and memory performance (CVLT; Pearson product moment correlation, *r*, two-tailed) scores.

We also performed bivariate correlation analyses (Pearson Product moment correlation, *r*, two-tailed) between the functional connectivity values and the medication doses computed according to the method by [32] and with the years on medication.

## 4. Conclusions

In the current study, BD patients showed altered intrinsic functional connectivity within the auditory/temporal cortex and in a fronto-temporal pathway. The diminished FC within temporal cortex areas might reflect a neural basis of performance deficits in verbal episodic memory. Furthermore, the increased fronto-temporal connectivity might serve as a compensatory pathway to maintain the recruitment of temporal cortex areas which are crucial for auditory speech perception. The correlations between language-related cognitive performance and functional alterations within the auditory network did not reach the level of statistical significance. Nonetheless, deficits in cognitive performance and language-related symptoms might be related to these underlying neuronal abnormalities as current pathophysiological models of BD show.

Although patients showed significantly more depressive symptoms than controls, all BD patients who underwent measurements of brain activity at rest were at remission stage. Thus, it can be hypothesized that abnormal patterns of functional connectivity within the auditory cortex, as well as an altered fronto-temporal functional connectivity may display trait markers of BD which are present even in symptom-free phases of the illness.
